# Chloroplast genome analysis and evolutionary insights in the versatile medicinal plant *Calendula officinalis* L.

**DOI:** 10.1038/s41598-024-60455-2

**Published:** 2024-04-26

**Authors:** Ningyun Zhang, Kerui Huang, Peng Xie, Aihua Deng, Xuan Tang, Ming Jiang, Ping Mo, Hanbin Yin, Rongjie Huang, Jiale Liang, Fuhao He, Yaping Liu, Haoliang Hu, Yun Wang

**Affiliations:** Agricultural Products Processing and Food Safety Key Laboratory of Hunan Higher Education, Hunan Provincial Key Laboratory for Molecular Immunity Technology of Aquatic Animal Diseases, College of Life and Environmental Sciences, Hunan University of Arts and Science, Changde, Hunan China

**Keywords:** *Calendula officinalis*, Chloroplast genome, Codon usage bias, Evolution, Adaptation, Genetics, Genome

## Abstract

*Calendula officinalis* L.is a versatile medicinal plant with numerous applications in various fields. However, its chloroplast genome structure, features, phylogeny, and patterns of evolution and mutation remain largely unexplored. This study examines the chloroplast genome, phylogeny, codon usage bias, and divergence time of *C. officinalis*, enhancing our understanding of its evolution and adaptation. The chloroplast genome of *C. officinalis* is a 150,465 bp circular molecule with a G + C content of 37.75% and comprises 131 genes. Phylogenetic analysis revealed a close relationship between *C. officinalis*, *C. arvensis*, and *Osteospermum ecklonis*. A key finding is the similarity in codon usage bias among these species, which, coupled with the divergence time analysis, supports their close phylogenetic proximity. This similarity in codon preference and divergence times underscores a parallel evolutionary adaptation journey for these species, highlighting the intricate interplay between genetic evolution and environmental adaptation in the Asteraceae family. Moreover unique evolutionary features in *C. officinalis*, possibly associated with certain genes were identified, laying a foundation for future research into the genetic diversity and medicinal value of *C. officinalis*.

## Introduction

*Calendula officinalis* L., a short-lived annual herbaceous species of the genus *Calendula* in the Asteraceae family, garners global recognition for its ubiquity and resilience. Predominantly located in the United States and Europe, it thrives in sunlit or partially shaded environments, necessitating minimal cultivation and management^1^. The plant stands 12–30 inches tall, characterized by its yellow to orange hermaphrodite flowers usually 2–3 inches in diameter, which bloom in a head-shaped inflorescence^2^. The leaves of *C. officinalis* are oblanceolate, alternate, sessile, and bright green, with stems adorned with umbel-like branches. The plant yields a curved, ring-shaped, and sickle-shaped achene^2^. The European Union has funded multiple research projects focused on *C. officinalis* due to its multifaceted role. Its diverse colors and aroma make it a favored decorative plant, and its bioactive compounds—carotenoids, saponins, amino acids—have found significant applications in chemical and pharmacological domains, offering anti-inflammatory, anti-viral, anti-genotoxic properties among others^2,3^. Despite its extensive utility and recognition as a versatile medicinal plant, *C. officinalis* L. remains a subject of scientific curiosity, particularly regarding its genetic makeup and evolutionary history. Previous studies have laid the groundwork by identifying its pharmacological benefits and some aspects of its bioactive compounds^2,3^. However, research into its chloroplast genome structure, phylogenetic relationships, and evolutionary dynamics has been notably sparse. This gap signifies a substantial opportunity to deepen our understanding of *C. officinalis*'s genetic underpinnings and evolutionary trajectory. Consequently, a comprehensive understanding of the chloroplast genome structure, phylogeny, and evolutionary mutation patterns of *C. officinalis* remains elusive.

Chloroplasts, critical plant organelles, govern photosynthesis, biosynthesis, and carbon sequestration^4^. These organelles possess an independent genetic system from the nuclear genome, and since the first chloroplast genome from *Nicotiana tabacum* was sequenced, the structure and function of chloroplast genomes have been progressively elucidated^5^. A typical chloroplast genome measures between 100 to 200 kb and has a four-part structure encompassing the large-single copy (LSC) region, the small-single copy (SSC) region, and two inverted repeat regions (IR)^6^. Chloroplast genome plays a crucial role in elucidating the evolutionary dynamics and phylogenetic relationships of plant species. By analyzing chloroplast DNA, particularly its conserved and variably evolving noncoding regions, researchers have gained insights into plant diversity, evolutionary rates, and lineage-specific evolutionary patterns^7–9^.

Codons form the fundamental link between nucleic acids and proteins, and synonymous codons, barring methionine and tryptophan, encode identical amino acids^10,11^. Codon usage bias, a phenomenon prevalent across organisms, refers to the variability in the frequency of synonymous codons coding for the same amino acid^12^. This bias not only mirrors the species' or genes' origin, evolution, and mutation patterns but also significantly impacts gene function and protein expression^13^. Despite previous studies focusing on the codon usage bias in nuclear genomes^14,15^, the chloroplast genomes' genetic code varies from the standard genetic code^16^, thereby necessitating an analysis of the codon usage bias in the chloroplast genomes. High-throughput sequencing technologies have facilitated the sequencing of numerous plant chloroplast genomes, and over two thousand have been deposited in GenBank at the National Center for Biotechnology Information (NCBI), thereby bolstering systematic evolutionary research based on chloroplast genome codon analysis. Most plant species' codon usage bias based on chloroplast genomes has been analyzed, and their phylogenetic status, evolution, and mutation patterns have been well-delineated. For instance, multiple species of the *Oryza* and *Gynostemma* genera have had their phylogenetic status, evolution, and mutation patterns elucidated through phylogenetic analysis and codon usage bias analysis based on chloroplast genomes.

In the present investigation, we characterized the chloroplast genome of *C. officinalis* and performed a comprehensive phylogenetic analysis anchored on this genome. Furthermore, we conducted a detailed exploration of the codon usage bias in *C. officinalis* and its closely related species *Calendula arvensis* and *Osteospermum ecklonis*. This approach facilitated our understanding of the plant's genomic attributes, evolutionary adaptation mechanisms, and phylogenetic positioning.

## Materials and methods

### Plant materials and genome sequencing

**Figure 1:**
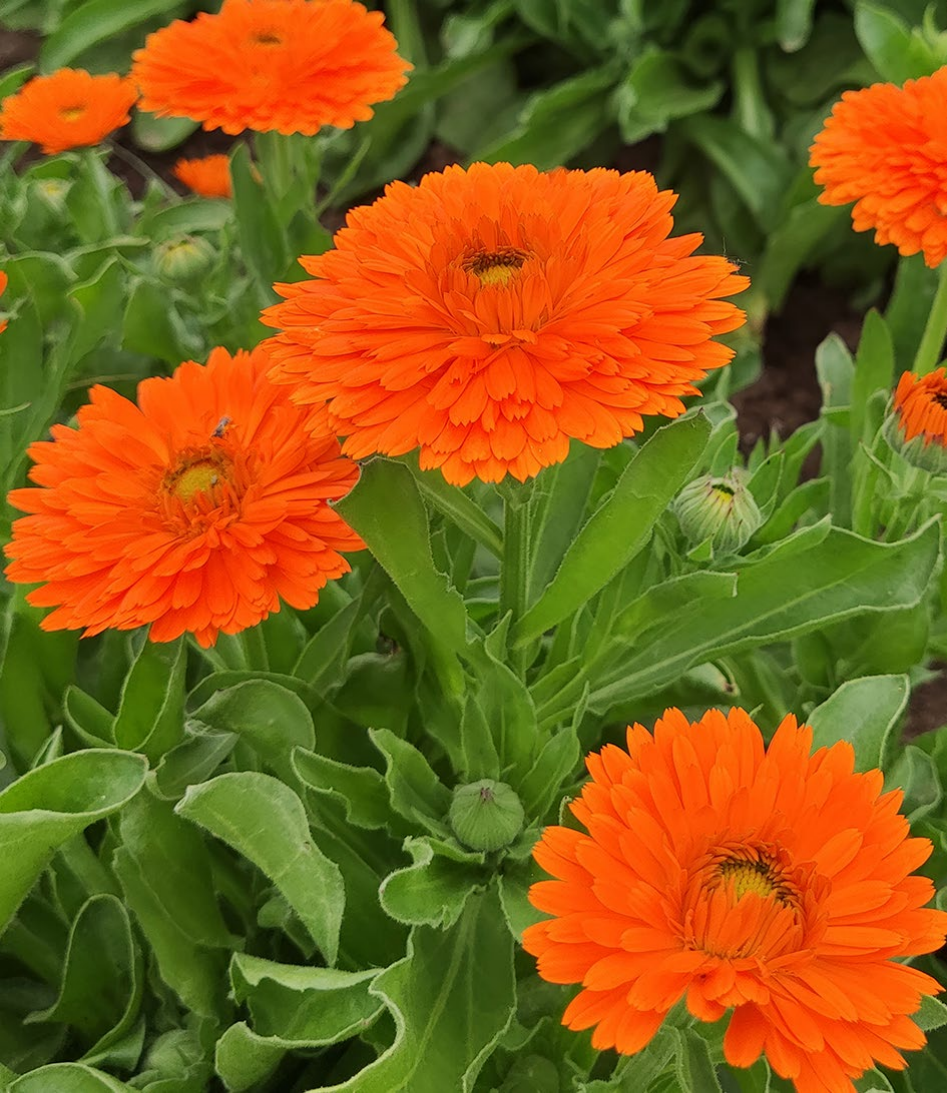
The picture of the collected sample of *Calendula officinalis*. The picture is self-taken nearby the Changde Vocational Technical College, Changde, Hunan province, China (N29°02′29.74", E111°38′05.31", 34 m). In the picture, *C. officinalis* is the plant 2–30 inches tall, with flowers usually 2–3 inches in diameter and a head-shaped inflorescence; the flowers are yellow to orange in color and hermaphrodite with tubular-shaped florets; The leaves are oblanceolate, alternate, sessile, bright green, and usually about 4 inches long; Stems with umbel-like branches; as well as curved, ring-shaped, and sickle-shaped achene. Y.W., H.H., and K.H. identified the plant.

Fresh leaves were picked from *C. officinalis* (Fig. 1) planted near the Changde Vocational Technical College, Changde, Hunan province, China (N29°02′29.74", E111°38′05.31", 34 m). The voucher specimens were well placed at the College of Life and Environmental Sciences, Hunan University of Arts and Sciences (Contact Person: Kerui Huang, huangkerui008@163.com, voucher number JZH007).

The library was constructed using the DNAsecure Plant Kit (TIANGEN Biotech Co., Ltd., Beijing) and the sequencing was performed on an Illumina HiSeq 2500 platform (San Diego, CA), both outsourced to Shanghai Personalbio Technology Co., Ltd. (China).

### Chloroplast genome assembling and annotation

After filtering out the low-quality reads using fastp^17^, 81,419,412 clean reads were retained for further analysis. The chloroplast genome of *C. officinalis* was de novo assembled using GetOrganelle v1.7.5^18^ with parameters set as -R 15 -k 21,45,65,85,105 -F embplant_pt. Subsequently, the assembled chloroplast genome was annotated using CPGAVAS2^19^ with default settings, and a circular genome map was visualized using CPGView (http://www.1kmpg.cn/cpgview/).

### Phylogenetic analysis

A total of 44 chloroplast genomes closely related to *C. officinalis*, along with 2 outgroups, were downloaded from GenBank for phylogenetic analysis. Among them, 74 protein-coding genes shared by all genomes were screened out for subsequent analysis. Sequence alignment of each gene was performed separately using MAFFT v7.313^20^. Gblocks 0.91b was then utilized to remove poorly aligned regions of each gene. The filtered gene sequences were concatenated head-to-tail into supergenes^21^. Maximum likelihood phylogenies were generated using IQ-TREE v1.6.12^22^. The TVM + F + I + G4 model was selected based on the Bayesian Information Criterion (BIC) in ModelFinder. This process was further strengthened with 5000 ultrafast bootstrap replications for robust statistical support along with Shimodaira-Hasegawa-like approximate likelihood ratio test.

### Codon usage bias analysis and IR border analysis

In this study, the chloroplast genome sequences of *C. officinalis*, *C. arvensis*, and *O. ecklonis* were used to analyze codon usage bias. Coding sequences (CDS) were meticulously screened to meet specific criteria: multiples of three in base count, sequence length ≥ 300 bp, inclusion of only A, T, C, G bases, presence of start (ATG) and stop codons (TAG, TGA, TAA), and absence of internal stop codons and duplicate sequences, retaining 53 CDS for each species. Using CodonW and CUSP online software, metrics such as ENc, RSCU, CAI, CBI, Fop, and GC content were calculated. Codons for Met, Trp, and stop codons were excluded. Analyses including ENc-plot, PR2-plot, neutrality plot^23,24^, and correspondence analysis based on RSCU values^25,26^ were conducted, assessing the influence of mutation pressure and natural selection on codon usage bias.

The comparative analysis of the boundaries separating the IRs, SSC, and LSC regions within chloroplast genomes was conducted utilizing the online tool IRscope, which is available at https://irscope.shinyapps.io/irapp (accessed on May 29, 2024).

### Divergence time estimation

The divergence times for the species included in our phylogenetic analysis were estimated using the Markov chain Monte Carlo (MCMC) approach implemented in the PAML software package, specifically utilizing its MCMCtree program^27^. The optimal phylogenetic tree topology for our dataset was determined using IQ-TREE.

For calibrating the molecular clock, we incorporated three fossil-based calibration points derived from previous studies^27–34^. These calibration points were as follows: (F1) between 22.7 and 38.8 million years ago (Ma), (F2) between 17.4 and 44.7 Ma, and (F3) between 1.13 and 33.48 Ma. These points were strategically chosen to constrain each corresponding node in the phylogenetic tree.

Our analysis employed the independent rates model (IRM), which assumes a lognormal distribution for rate variation among lineages. The HKY85 model was selected for nucleotide substitution, with the alpha parameter for gamma-distributed rate variation across sites set at 0.5. The birth–death process model was used to establish priors for node ages within the phylogenetic tree. We adhered to the default settings for this model, with the parameters λ (birth rate) and μ (death rate) both set to 1, and the sampling proportion (s) set to 0. For the MCMC analysis, posterior probabilities of the parameters were estimated. The initial 10% of trees generated were discarded as burn-in to ensure sampling from a stationary distribution. Subsequent trees were sampled every 10 iterations, culminating in a total of 10,000 sampled trees for the final analysis.

### Statement of permission and compliance

We confirm that *Calendula officinalis* materials used in this study were collected with permission from the parterre of Changde Vocational Technical College. The collection complied with all relevant local legislation, and appropriate permissions were granted before the samples were collected. All experiments and field studies on *Calendula officinalis* in this research complied with local legislation and were carried out in accordance with relevant institutional, national, and international guidelines and legislation. The experimental protocols were approved by the relevant ethics committee.

## Results

### The chloroplast feature of *C. officinalis*

**Figure 2:**
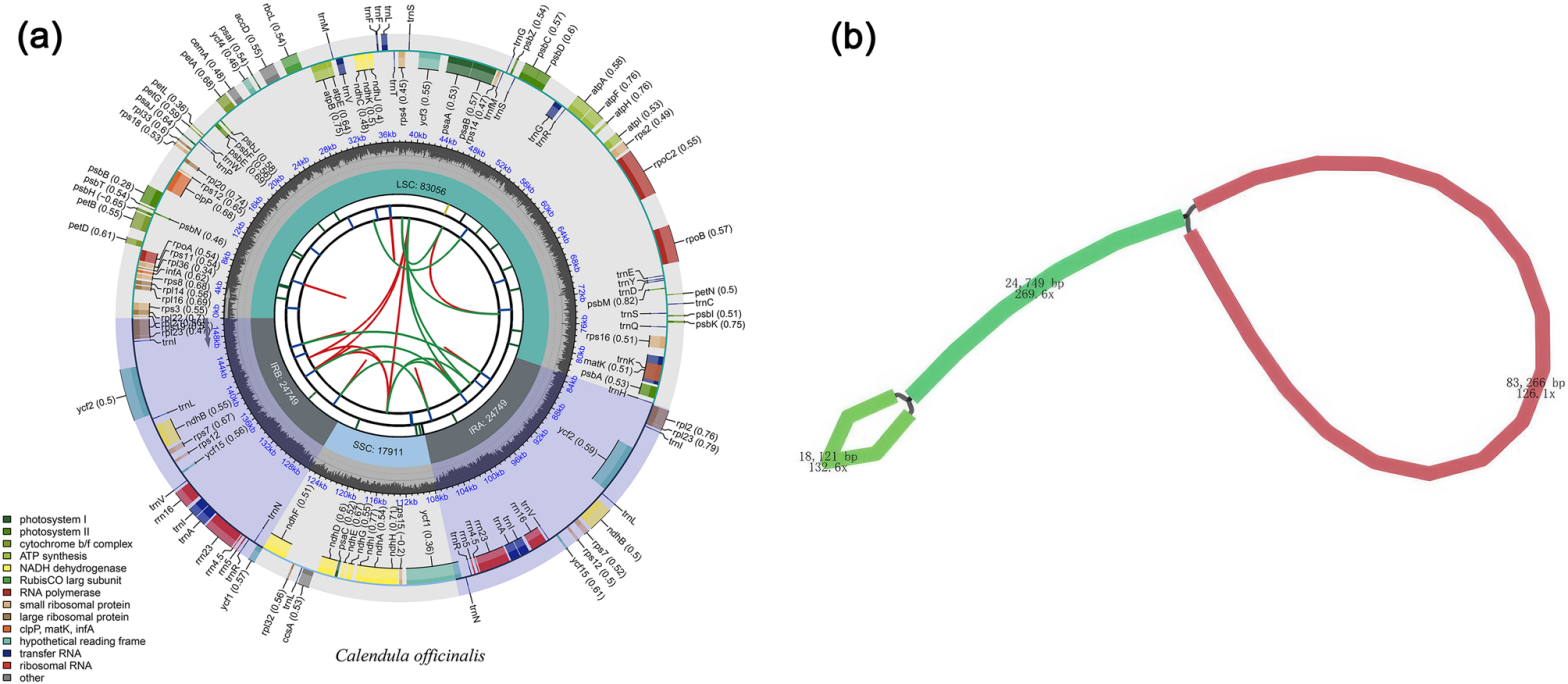
The gene map and the coverage depth schematic of the *Calendula officinalis* chloroplast genome. (**a**) The gene map. The center of the figure represents the genome, and the various tracks surrounding it represent different features. The first track shows the dispersed repeats, while the second track displays the long tandem repeats as short blue bars. The third track indicates the short tandem repeats or microsatellite sequences as short bars with different colors. The fourth track shows the small single-copy (SSC), inverted repeat (Ira and Irb), and large single-copy (LSC) regions. The GC content along the genome is plotted on the fifth track. Finally, the genes are displayed on the sixth track, and the optional codon usage bias is displayed in the parenthesis after the gene name. (**b**) The schematic representation of the coverage depth for the entire chloroplast genome of *Calendula officinalis* using Bandage. The numbers indicate the depths and length of different regions.

The chloroplast genome of *C. officinalis* is a circular molecule of 150,465 bp in length (Fig. 2a), which consists of four parts: a large single-copy region (LSC) with a length of 83,056 bp; a small single-copy region (SSC) with a length of 17,911 bp; and two inverted repeat regions (IRs) of 24,749 bp (Fig. 2). The G + C content of the whole chloroplast was 37.75%, and the IRs were 43.11%, which was higher than that of the LSC and the SSC regions (35.84% and 31.81%). The schematic representation of the entire chloroplast genome of *Calendula officinalis* is depicted in Fig. 2b, and Fig. S1 illustrates the uniform mapping depth across the entire genome, indicating an absence of heteroplasmy. The genome contains 131 genes, including 86 protein-coding genes, eight rRNA genes, and 37 tRNA genes (Fig. 2), the cis-splicing genes and trans-splicing gene *rps12* in the chloroplast genome of *Calendula officinalis* can be found in Fig. S2.

### Phylogenetic analysis

**Figure 3:**
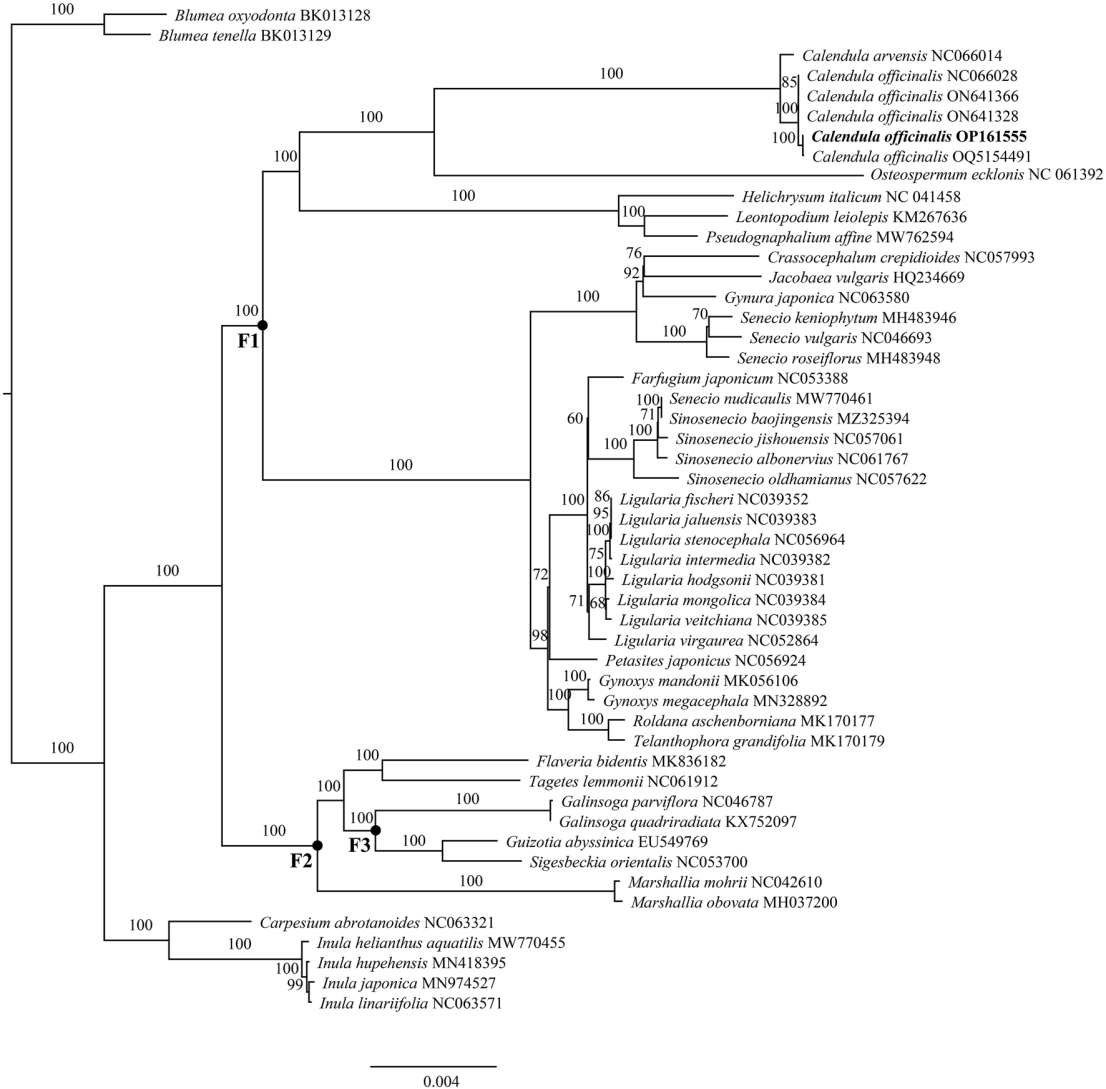
Maximum-likelihood (ML) tree of *Calendula officinalis* and 44 related species along with two outgroups using the IQ-Tree based on 74 protein-coding genes shared by all genomes. Bootstrap values are shown next to the nodes. The following sequences were used: *Roldana aschenborniana* MK170177, *Telanthophora grandifolia* MK170179, *Crassocephalum crepidioides* NC057993, *Gynura japonica* NC063580 *Jacobaea vulgaris* HQ234669, *Senecio keniophytum* MH483946, *Senecio vulgaris* NC046693, *Senecio roseiflorus* MH483948, *Blumea oxyodonta* BK013128, *Blumea tenella* BK013129, *Carpesium abrotanoides* NC063321, *Inula helianthus aquatilis* MW770455, *Inula hupehensis* MN418395, *Inula japonica* MN974527, *Inula linariifolia* NC063571, *Flaveria bidentis* MK836182, *Tagetes lemmonii* NC061912, *Galinsoga parviflora* NC046787, *Galinsoga quadriradiata* KX752097, *Guizotia abyssinica* EU549769, *Sigesbeckia orientalis* NC053700, *Marshallia mohrii* NC042610, *Marshallia obovata* MH037200, *Helichrysum italicum* NC041458, *Leontopodium leiolepis* KM267636, *Pseudognaphalium affine* MW762594, *Osteospermum ecklonis* NC061392, *Farfugium japonicum* NC053388, *Senecio nudicaulis* MW770461, *Sinosenecio baojingensis* MZ325394, *Sinosenecio jishouensis* NC057061, *Sinosenecio albonervius* NC061767, *Sinosenecio oldhamianus* NC057622, *Ligularia virgaurea* NC052864, *Ligularia fischeri* NC039352, *Ligularia stenocephala* NC056964, *Ligularia jaluensis* NC039383, *Ligularia intermedia* NC039382, *Ligularia mongolica* NC039384, *Ligularia veitchiana* NC039385, *Ligularia hodgsonii* NC039381, *Syneilesis aconitifolia* NC061374, *Petasites japonicus* NC056924, *Gynoxys mandonii* MK056106, *Gynoxys megacephala* MN328892. Calibration points F1, F2, and F3, used for divergence time analysis, are marked on the tree with bold dots.

Based on the chloroplast genome of *C. officinalis*, the Maximum-likelihood (ML) tree was constructed (Fig. 3) using 74 protein-coding genes, which helps to determine *C. officinalis*’ phylogenetic status. Phylogenetic analysis indicates that, broadly, the support values for each clade of the phylogenetic tree exceed 50%, with the majority reaching 100%, demonstrating the reliability of our phylogenetic tree (Fig. 3). Further, *C. officinalis* and *C. arvensis* were within one clade with a support of 100%, and also, from a local point of view, the relationship between *C. officinalis*, *C. arvensis,* and *O. ecklonis* was very close, although *O. ecklonis* does not belong to the *Calendula* genus*,* which is consistent with the previous study^35^. Interestingly, *Crassocephalum crepidioides*, *Gynura japonica*, *Jacobaea vulgaris*, and *Seneico vulgaris* were found to be more closely related to *C. officinalis* in our study (Fig. 3), compared to previous reports. This close relationship represents a novel finding, likely attributed to the differences in sequence data and phylogenetic methods employed in this study, as the protein-coding genes extracted from complete chloroplast genomes contain richer information compared to previous marker genes used.

### Codon composition analysis

The codon composition for CDS of the three species (*Calendula officinalis*, *Calendula arvensis*, and *Osteospermum ecklonis*) was analyzed (Table 1), and the GC content of chloroplast-encoded genes in the three Asteraceae plants is 38.49%, 38.50%, and 38.54%, respectively. The GC content varies at different positions, with the first, second, and third positions in the codons all having a GC content below 50%. The highest content is at the first base, and the lowest content is at the third base, showing a trend of GC1 > GC2 > GC3. This indicates that the chloroplast genome sequences of the three Asteraceae plants are rich in A/T bases, particularly at the third position of the codons. the ENc values of chloroplasts in the three Asteraceae plants (*C. officinalis*, *C. arvensis*, and *O. ecklonis*) are 37.6959.17, 38.7459.17, and 39.1 ~ 58.98, with average values of 47.34, 47.41, and 47.65, respectively. All of these values are significantly greater than 35, indicating that the codon usage bias in the chloroplast genomes is relatively weak.

**Table 1:** GC content and effective number of codons (ENc) in *Calendula officinalis*, *Calendula arvensis*, and *Osteospermum ecklonis.*

Species	GeneBank ID	GCall	GC1	GC2	GC3	GC3s	ENc
*Calendula officinalis*	OP161555.1	38.49	47.18	39.96	28.34	25.21	47.34
*Calendula arvensis*	NC066014.1	38.50	47.28	39.97	28.27	25.15	47.41
*Osteospermum ecklonis*	NC061392.1	38.54	47.19	39.82	28.61	25.55	47.65

GCall: Overall guanine-cytosine (GC) content in the species' genomic sequence (percentage); GC1: GC content at the first codon position in the species' genomic sequence (percentage); GC2: GC content at the second codon position in the species' genomic sequence (percentage); GC3: GC content at the third codon position in the species' genomic sequence (percentage); GC3s: GC content at synonymous third codon positions in the species' genomic sequence (percentage); ENc: Effective number of codons.

### Codon usage bias analysis

**Figure 4:**
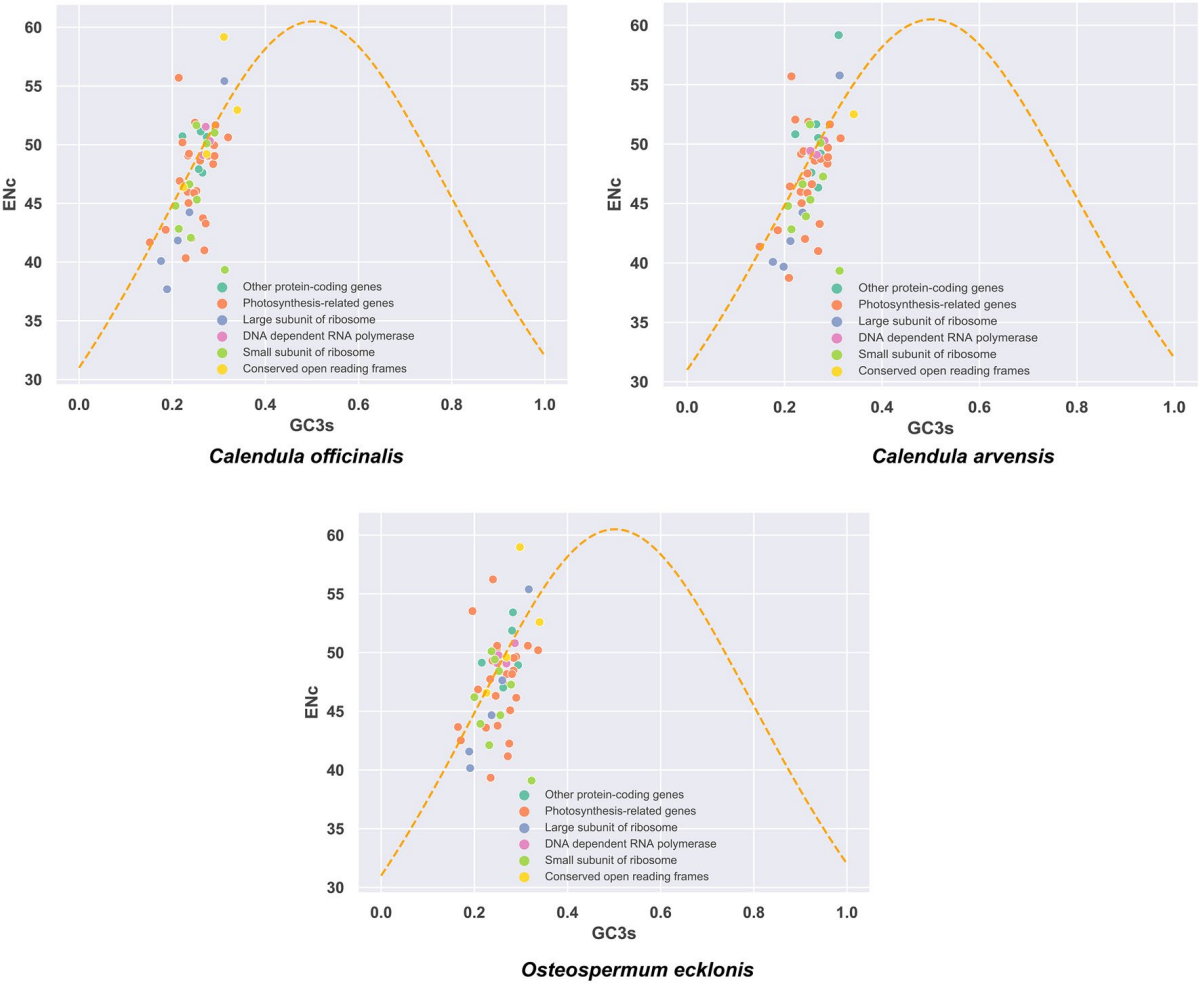
Effective number of codons (ENc) of each coding gene against GC3s for the chloroplast genome of *Calendula officinalis*, *Calendula arvensis*, and *Osteospermum ecklonis*. The scatterplot displays the relationship between the effective number of codons (ENc) and GC content at the third codon position (GC3s) for each coding gene in the chloroplast genomes of the three species. Different colors represent genes of different types.

By using GC3 and ENc as the X-axis and Y-axis, respectively, for the c analysis, the influence of nucleotide composition on codon preference can be detected. When genes are distributed along the standard curve or near it, it indicates that the codon preference of the gene is affected only by mutations. However, when genes fall far below the standard curve, it indicates that the codon preference of the gene is affected by selection. From the result of this study, it can be observed that the ENc-plot diagrams (Fig. 4) of the chloroplast genomes of the three Asteraceae plants (*C. officinalis*, *C. arvensis*, and *O. ecklonis*) are similar, some genes are distributed along the standard curve or close to it, indicating that their codon preference is mainly influenced by nucleotide mutations. However, some other genes deviate from the standard curve, suggesting that nucleotide mutations are not the main factor affecting their codon preference and that they may be affected by other factors such as natural selection (Fig. 4). In addition to the similarity, there are a few differences, for example, photosynthesis-related genes of *C. officinalis* and *C. arvensis* are mainly concentrated near the standard curve, while those of *O. ecklonis* deviate below slightly, indicating that the photosynthesis-related genes of *O. ecklonis* might be more influenced by selection, while those of *C. officinalis* and *C. arvensis* are primarily affected by mutations.

**Figure 5:**
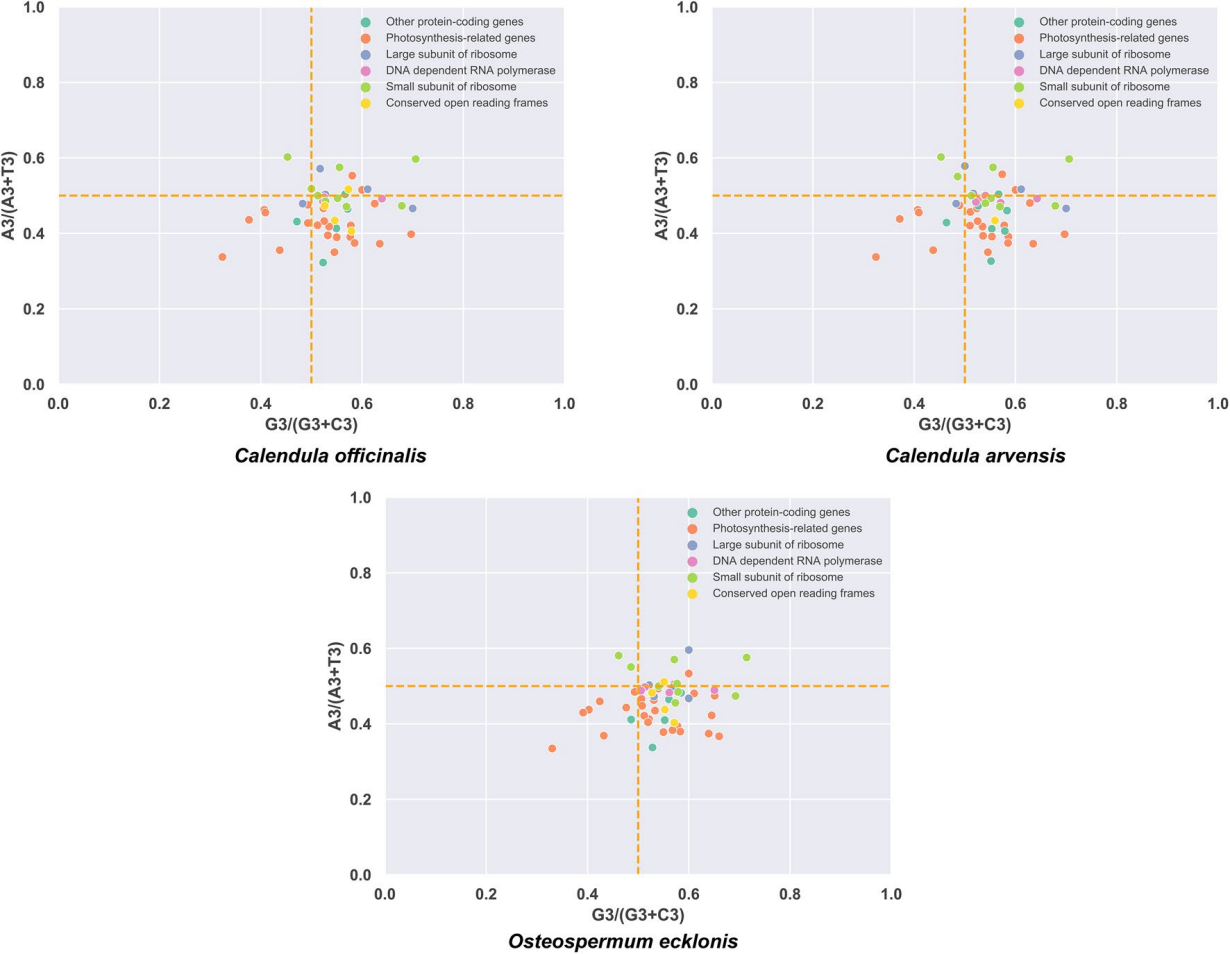
The parity rule 2 (PR2) bias plots for genes of the chloroplast genome of *Calendula officinalis*, *Calendula arvensis*, and *Osteospermum ecklonis*. The scatterplot displays the relationship between A3/(A3 + T3) and G3/(G3 + C3) for each coding gene in the chloroplast genomes of the three species, representing the parity rule 2 (PR2) bias. Different colors represent genes of different types.

Parity rule 2 plots (PR2 plot) were generated respectively for the three Asteraceae plants (*C. officinalis*, *C. arvensis*, and *O. ecklonis*) using the chloroplast's protein-coding genes (Fig. 5). It can be easily noticed that all three plots were with great similarity. Firstly, the majority of their coordinate points are not uniformly distributed across the four regions but are mainly concentrated in the region where G3/(G3 + C3) > 0.5 and A3/(A3 + T3) < 0.5 (Fig. 5). Then, genes of the small subunit of ribosome of all three species tend to use A more, while Photosynthesis-related genes lean towards using T. However, overall, the usage frequency of the third base T in the codon is higher than A, and the usage frequency of G is higher than C. If codon usage bias were solely caused by nucleotide mutations, the usage frequencies of A/T and G/C should be equal. Therefore, the PR2-plot analysis results, combined with the ENc-plot analysis, indicate that the codon usage bias in the chloroplast genomes of the three Asteraceae plants is formed by the combined effects of nucleotide mutations and natural selection. The similarity of the PR2-plot analysis results reflects the similarity of their phylogenetic relationships.

**Figure 6:**
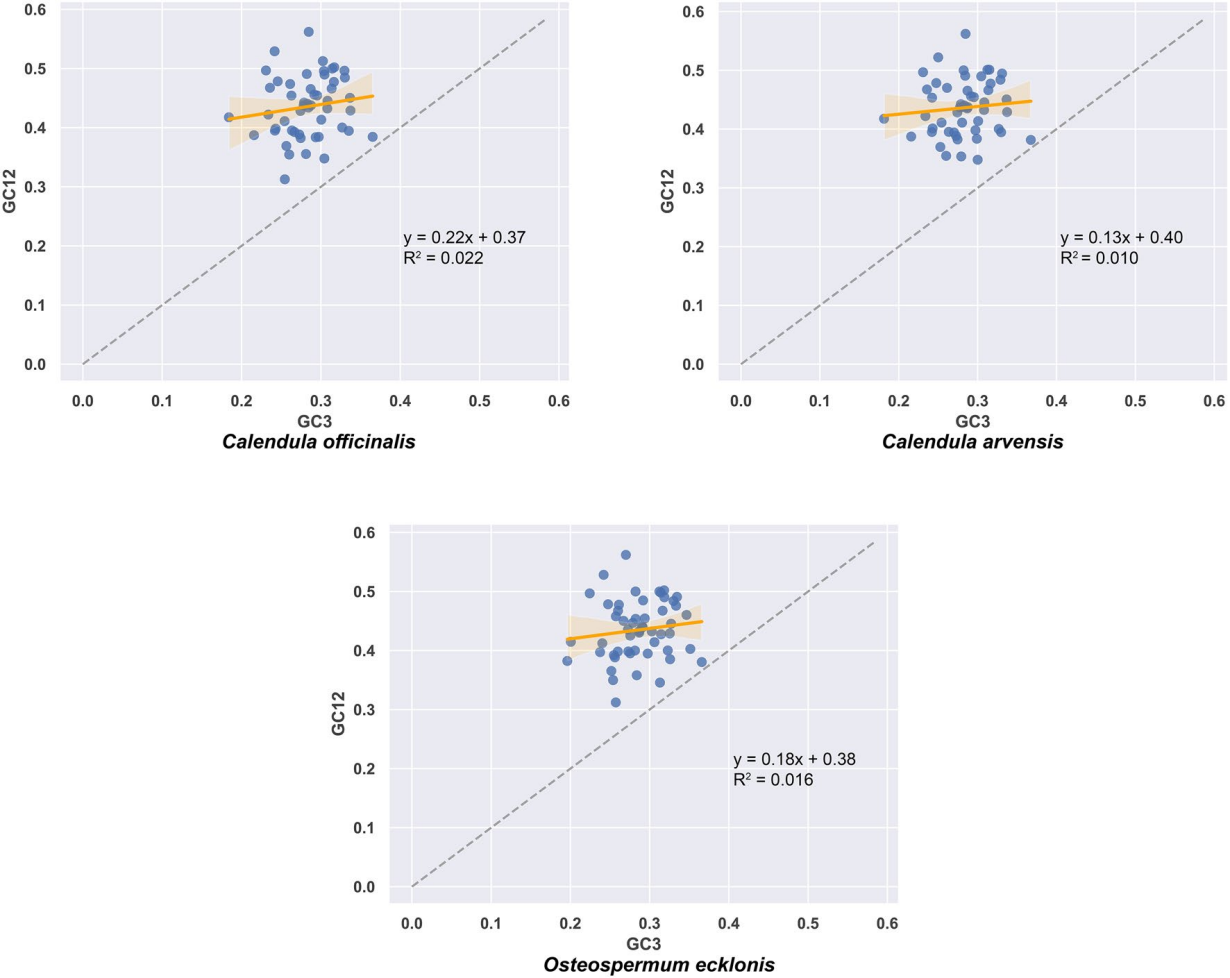
Neutrality plot for genes of the chloroplast genome of *Calendula officinalis*, *Calendula arvensis*, and *Osteospermum ecklonis*. The scatterplot displays the correlation between the average GC content at the first and second codon positions (GC12) and the GC content at the third codon position (GC3) for each coding gene in the chloroplast genomes of the three species.

The correlation between codon GC12 and GC3 for the chloroplast genomes of the three Asteraceae plants (*C. officinalis*, *C. arvensis*, and *O. ecklonis*) is quite familiar as the Neutrality plot showed (Fig. 6). The codon GC12 values of the three plants' chloroplast genomes are distributed between 27.85 and 58.88, while GC3 values are distributed between 18.40 and 36.74, indicating that the frequency of using A/T at the third codon position is higher than G/C (Fig. 6). The slope of the regression line fitted with GC12 and GC3 ranges from 0.13 to 0.22, with R^2^ > 0, suggesting a positive correlation between G12 and G3 values. However, the two-tailed test did not reach significant levels (P > 0.05) for all three species, indicating that the mutation patterns of the first and second bases are different from the third base, and the codon usage bias is more affected by natural selection than by nucleotide mutations (Fig. 6). Additionally, the regression coefficient of *O. ecklonis* is closest to 0, indicating that its chloroplast genome codon preference is most influenced by natural selection, while *C. officinalis* has the furthest regression coefficient from 0, suggesting that its chloroplast genome codon preference is least influenced by natural selection compared to the other two Asteraceae plants.

**Figure 7:**
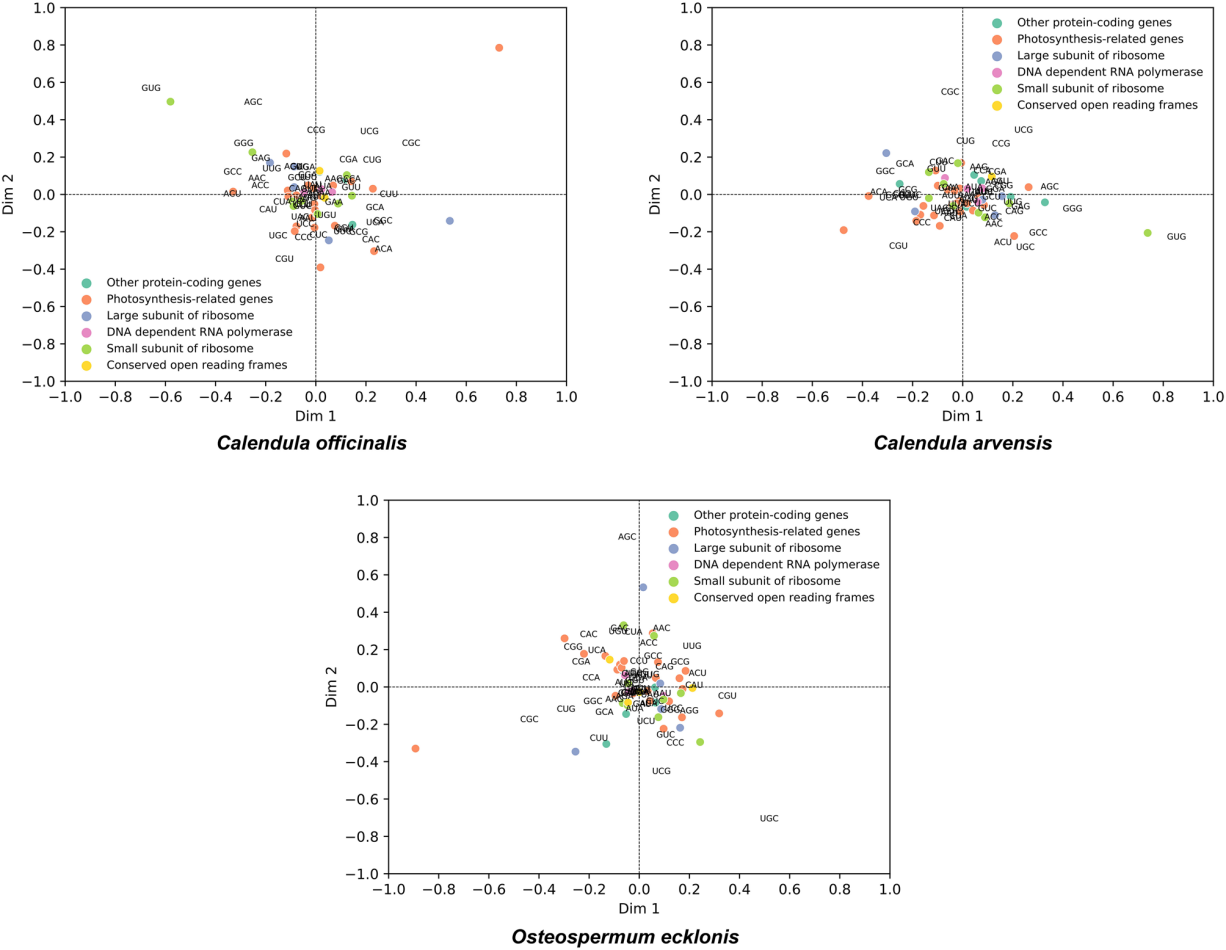
Correspondence analysis of the RSCU values of the chloroplast genome of *Calendula officinalis*, *Calendula arvensis*, and *Osteospermum ecklonis*. Each point on the plot corresponds to the coordinates on the first and second-principal axes, representing the distribution of the genes based on their relative synonymous codon usage (RSCU) values. The codons also distribute in the plot.

As the result of the correspondence analysis, the genes (CDS sequences) of the chloroplast genomes of the three Asteraceae plants (*C. officinalis*, *C. arvensis*, and *O. ecklonis*) are distributed on the figure with the first major factor axis as the x-coordinate and the second major factor axis as the y-coordinate (Fig. 7). The origin represents the average RSCU values of all genes relative to the first axis and the second axis. The sum of the proportions of the total variation accounted for by the first four principal factor axes in the three Asteraceae plants are 34.80%, 34.69%, and 33.66%, respectively (Fig. 7). The proportion of the total variation accounted for by the first principal factor axis is 9.93%, 9.93%, and 9.23%, respectively (Fig. 7), indicating that the first axis contributes the most to the variation, and the contribution of the remaining factor axes decreases successively. This again suggests that the formation of codon usage bias characteristics in the chloroplast genes of the three Asteraceae plants is not influenced by a single factor but is the result of the combined action of multiple factors.

To explore the factors affecting the distribution of each gene in the correspondence analysis plot of the chloroplast genomes of the three Asteraceae plants, correlation analysis between the first axis and GC3s, ENc, CAI, CBI, and Fop, respectively was performed. As can be seen from Table 2, the GC3s and CAI values of *C. officinalis* are significantly correlated with the first axis (P < 0.05); the GC3s and CAI values of *C. arvensis* are also highly significantly correlated with the first axis (P < 0.05), and the ENc value is significantly correlated with the first axis; the ENc value of *O. ecklonis* is significantly correlated with the first axis (Table 2). It can be found that, when not considering the slope direction, the correlation between the first axis and various indicators of *C. officinalis* and *C. arvensis* is closer, while there is a larger difference with *O. ecklonis*. This pattern is consistent with that shown in the phylogenetic tree (Fig. 3), reflecting the similarity and differences among the three, which indicate that correspondence analysis may reveal the commonalities and subtle differences in codon usage bias among the three species, which may be an important characteristic reflecting the differences in their phylogenetic relationships, even if their relationships are relatively close.

**Table 2:** Correlations of ENc, GC3s, GC, CAI, and Fop with the first axis of correspondence analysis for *Calendula officinalis*, *Calendula arvensis*, and *Osteospermum ecklonis* respectively.

Features	*Calendula officinalis*	*Calendula arvensis*	*Osteospermum ecklonis*
ENc	− 0.173694761	0.339292696*	− 0.298674522*
GC3s	− 0.355259062*	0.386953196**	0.148086675
GC	0.027698715	− 0.051792803	0.117641069
CAI	0.323028467*	− 0.366083451**	− 0.016358954
Fop	0.174586161	− 0.197259938	0.062726022

The numerical values in the table represent correlation coefficients between the respective indices (ENc, GC3s, GC, CAI, and Fop) and the first axis of the corresponding analysis.

*ENc* effective number of codons, *GC3s* GC content at synonymous third codon positions in a species' genomic sequence (percentage), *GC* Guanine-Cytosine content, *CAI* codon adaptation index, *Fop* frequency of optimal codons.

An asterisk (*) indicates a significant correlation at P < 0.05, while two asterisks (**) denote a highly significant correlation at P < 0.01.

### Divergence time analysis

**Figure 8:**
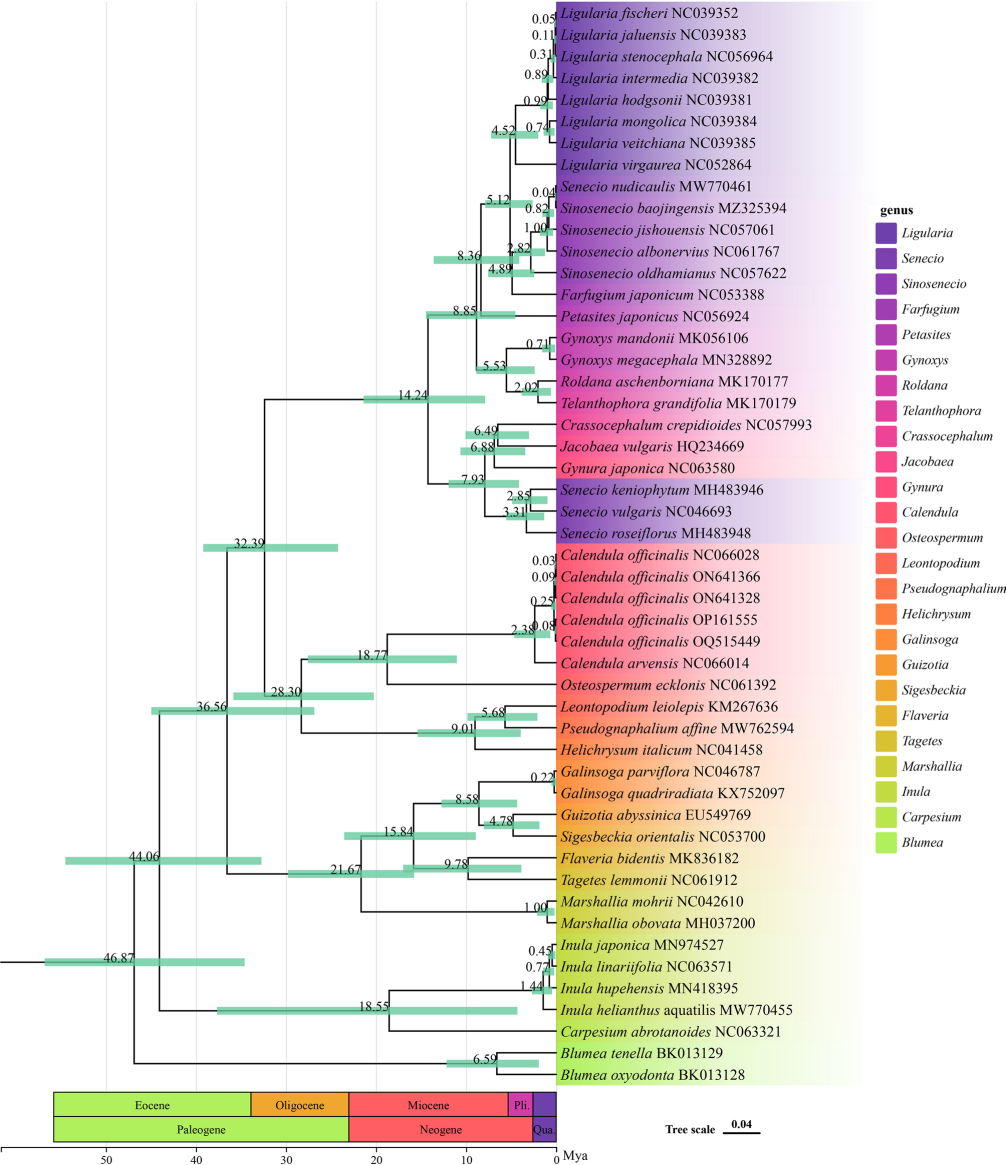
Divergence time estimation based on cp genome sequences. The divergence times are exhibited on each node, whereas the greed bars represent the 95% highest posterior density interval for each node age.

Our divergence time analysis, as illustrated in Fig. 8, indicates that the divergence of *C. officinalis* took place approximately 0.25 million years ago (Mya), situating it in the recent Quaternary period. Additionally, the genus *Calendula* is estimated to have originated around 2.38 Mya, also during the Quaternary. Furthermore, the common ancestor of *Calendula* and *Osteospermum* is traced back to roughly 18.77 Mya, placing this divergence squarely within the Miocene epoch of the Neogene period, an era known for significant environmental and climatic shifts that likely influenced their evolutionary paths.

**Figure 9:**
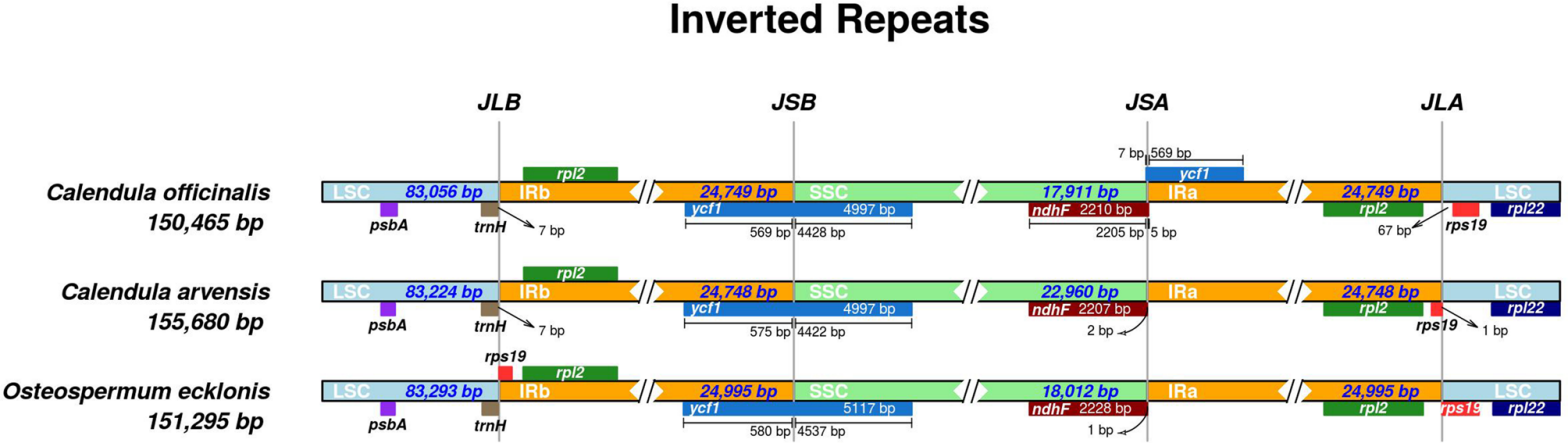
Comparison of boundaries between LSC, SSC, and IR regions among *Calendula officinalis*, *Calendula arvensis*, and *Osteospermum ecklonis*.

To further explore the subtle evolutionary differences among the chloroplast genomes of three species, we conducted a comparative analysis of the boundaries of the LSC, SSC, and IR regions across these species (Fig. 9). The result reveals a notable consistency across three species, primarily reflected in the genes adjacent to IR boundaries. Specifically, the genes near the JLA (junction of the LSC and the IRa region) consistently include *rpl2*, *rps19*, and *rpl22* across the species examined. Additionally, *psbA*, trnH*,* and *rpl22* genes are entirely located within the LSC region, while two copies of the *rpl2* gene are fully situated within IRa and IRb, respectively. The ycf1 gene spans the IRb and the SSC regions, positioned at the JSB junction.

Despite these overarching similarities, specific differences in gene placement and IR boundary dynamics are evident among the species. A notable distinction is observed in the placement of the *ycl1* gene, which spans the JSA junction (junction of the SSC and IRa region) exclusively in *C. officinalis*, with the majority of its sequence within IRa (extending 7 bp into the SSC), indicating a significant expansion/contraction event. This occurrence is not mirrored in the other two species. Furthermore, the *ndhF* gene in *C. officinalis* predominantly resides within the SSC, marginally spanning the JSA junction by 5 bp, whereas in the other species, it is completely contained within the SSC, showcasing a unique trait of* C. officinalis*.

In another aspect, the *rps19* gene is located entirely within the IRb near the JLB (junction of the IRb and the LSC region) and near the JLA in the LSC for *O. ecklonis*, while in *C. officinalis* and *C. arvensis*, it appears only once, situated near the JLA in the LSC and IRa, respectively (Fig. 9).

## Discussion

*C. officinalis* is a versatile plant with applications in various fields, including ornamentation, chemistry, and pharmacology. Despite its widespread use, its chloroplast genome structure, features, phylogeny, and patterns of evolution and mutation have remained largely unexplored. This study aims to address this knowledge gap by examining the chloroplast genome, phylogeny, and codon usage bias of *C. officinalis*, thereby enhancing our understanding of its evolution, adaptation, and potential uses.

The chloroplast genome of *C. officinalis* was found to be a 150,465 bp circular molecule, containing a large single-copy region (LSC), a small single-copy region (SSC), and two inverted repeat regions (IRs). The genome's G + C content is 37.75%, and it comprises 131 genes, including 86 protein coding, eight rRNA, and 37 tRNA genes. A Maximum-likelihood (ML) tree was constructed using 74 protein-coding genes to establish the phylogenetic status of *C. officinalis*. Phylogenetic analysis revealed that *C. officinalis* and *C. arvensis* form a clade with 100% support, and their relationship with *O. ecklonis* is close, in accordance with a previous study. The analysis also indicated a closer relationship between *C. officinalis* and four other species than previously reported^35^. This discrepancy could be attributed to differences in sequences and methods employed for phylogenetic analysis, warranting further investigation.

Codon usage bias is a vital element of evolution across diverse genomes, which is influenced by multiple biological factors including gene expression, gene length, tRNA abundance, mutation bias, and GC composition, as evidenced by a wealth of studies^36–42^. Nevertheless, it's the interplay between directional mutation pressure and natural selection that primarily governs codon usage bias across diverse organisms, forming the bedrock of interspecies and intragenomic codon usage disparities^43^. Plant genomes further demonstrate the complexity of codon usage bias; the nuclear gene codon preference is largely shaped by nuclear acid composition constraints, whereas in the realm of chloroplast and mitochondrial genomes, natural selection takes precedence^44,45^. The effective number of codons (ENc) is a common metric to quantify the degree of deviation in codon usage from random selection. Ranging in value from 20 to 61, ENc helps evaluate the strength of codon usage bias in genomes or genes. Smaller ENc values signify stronger codon preference, while larger values indicate weaker codon preference. Notably, when the ENc value is less than or equal to 35, the codon usage bias phenomenon is considered more significant.

In our research, we found that the chloroplast genome sequences of the three Asteraceae plants *C. officinalis*, *C. arvensis*, and *O. ecklonis* were rich in A/T bases, with ENc values ranging from 37.69 to 59.17, 38.74 to 59.17, and 39.1 to 58.98, and average values of 47.34, 47.41, and 47.65, respectively. As all these values are significantly greater than 35, this suggests that the codon usage bias in the chloroplast genomes of these species is relatively weak. Further analysis using ENc-plot (Fig. 4), PR2-plot (Fig. 5), Neutrality plot (Fig. 6), and correspondence analysis (Fig. 7) revealed that the codon usage bias in *C. officinalis*, *C. arvensis*, and *O. ecklonis* is a result of the combined effects of natural mutation and selection pressure. In addition, the codon usage bias patterns in these three species are highly similar, providing a robust explanation for their phylogenetic similarities. This finding suggests that these species may have been subjected to similar environmental conditions and selection pressures during their evolutionary process. This similarity in environmental conditions and selection pressures could also account for the close phylogenetic relationship between the species *O. ecklonis* and *C. officinalis* and *C. arvensis*. Moreover, we discovered that the correlation of the first axis of the correspondence analysis with GC3s, ENc, CAI, CBI, and Fop can effectively reflect the subtle differences in codon usage bias patterns among the three species. Interestingly, these differences are consistent with the results of the phylogenetic tree, indicating that this phenomenon is worthy of further study.

The divergence of the genus *Calendula* around 2.38 Ma (Fig. 8), within the Quaternary period, corresponds to a phase of Earth's history marked by intense climatic fluctuations. This period, characterized by repeated glacial and interglacial cycles^46,47^, would have imposed strong selective pressures on plant species, driving adaptive responses. The speciation of *C. officinalis* during this time suggests its evolutionary resilience and adaptability to changing environments. This aligns with our findings of weak codon usage bias in the chloroplast genome, indicative of a balanced selection-mutation dynamic possibly influenced by these environmental shifts.

The emergence of the common ancestor of *Calendula* and *Osteospermum* around 18.77 Ma (Fig. 8) in the Miocene epoch of the Neogene period coincides with significant global climate changes from warmer to cooler conditions^48^. The Miocene epoch, known for its extensive tectonic activities and consequent ecological shifts^49^, likely provided diverse niches and selective pressures that catalyzed speciation events. The similarity in codon usage bias patterns among *Calendula* and *Osteospermum* species provides further evidence of their phylogenetic relationship and shared evolutionary history. This similarity suggests a parallel adaptation route, possibly as a response to similar environmental pressures over time.

However, despite the many evolutionary similarities among the three species, our comparative analysis of the boundaries of the LSC, SSC, and IR regions highlights distinct differences in the expansion and contraction of the *ycl1* and *ndhF* genes in *C. officinalis*, compared to the other two species. In the evolutionary progression of angiosperms, the alteration, reduction, and enlargement of IR regions represent frequent events. Such changes often take place at the junctions between IRs and LSC and SSC, facilitating the movement of specific genes into either IR or single-copy regions^50^. The unique characteristics of these two genes may reflect the distinct nature of C. *officinalis* as a species that emerged relatively recently in evolutionary terms (0.25 Ma, compared to the divergence time of 18.77 Ma among these three species). This distinctiveness warrants further in-depth investigation to understand its evolutionary implications and the adaptive significance of these genomic features.

The insights gleaned from this research not only improve our understanding of the evolutionary relationships and adaptation mechanisms of *C. officinalis* and related species but also lay the groundwork for future investigations into the potential applications of these plants. Understanding the divergence time and evolutionary adaptations of *C. officinalis* opens avenues for exploring its potential applications in pharmacology and agriculture. Future research could delve deeper into how specific adaptations in the chloroplast genome have contributed to its medicinal and ornamental properties.

## Conclusion

In summary, our research provides a comprehensive analysis of the chloroplast genome, phylogenetic relationships, codon usage bias, and divergence time of *C. officinalis*. It highlights the close evolutionary kinship between *C. officinalis*, *C. arvensis*, and *O. ecklonis*, supported by similarities in codon usage patterns and divergence timelines. These findings suggest that shared environmental selection pressures have played a significant role in their evolutionary paths. Moreover, we have identified unique evolutionary features in *C. officinalis*, possibly associated with certain genes. Besides, our findings enhance the understanding of the genetic makeup of *C. officinalis*. This deeper genetic insight lays a foundation for future research into the genetic diversity and medicinal value of *C. officinalis*, potentially unlocking new avenues for exploiting its properties in pharmacology and agriculture. Our analysis not only advances knowledge of the *C. officinalis* genome but also sets the stage for exploring its genetic diversity and tapping into its vast medicinal potential, highlighting the importance of continued investigation into this valuable plant species.

### Supplementary Information

Supplementary Figures.

## Data Availability

The complete chloroplast genome sequence of *C. officinalis* has been deposited in the GenBank database under the accession number OP161555 (https://www.ncbi.nlm.nih.gov/nuccore/OP161555.1/). The associated BioProject and Bio-Sample numbers are PRJNA1019102 and SAMN37474090, respectively.
