# Supplementary material for: Chloroplast genome analysis and evolutionary insights in the versatile medicinal plant Calendula officinalis L
Source: Sci Rep. 2024 Apr 26;14:9662. doi: 10.1038/s41598-024-60455-2 (PMC11053094; doi:10.1038/s41598-024-60455-2)
Supplement: Supplementary file 1 — Supplementary Figures. [file 41598_2024_60455_MOESM1_ESM.docx]

**Supplemental Material**


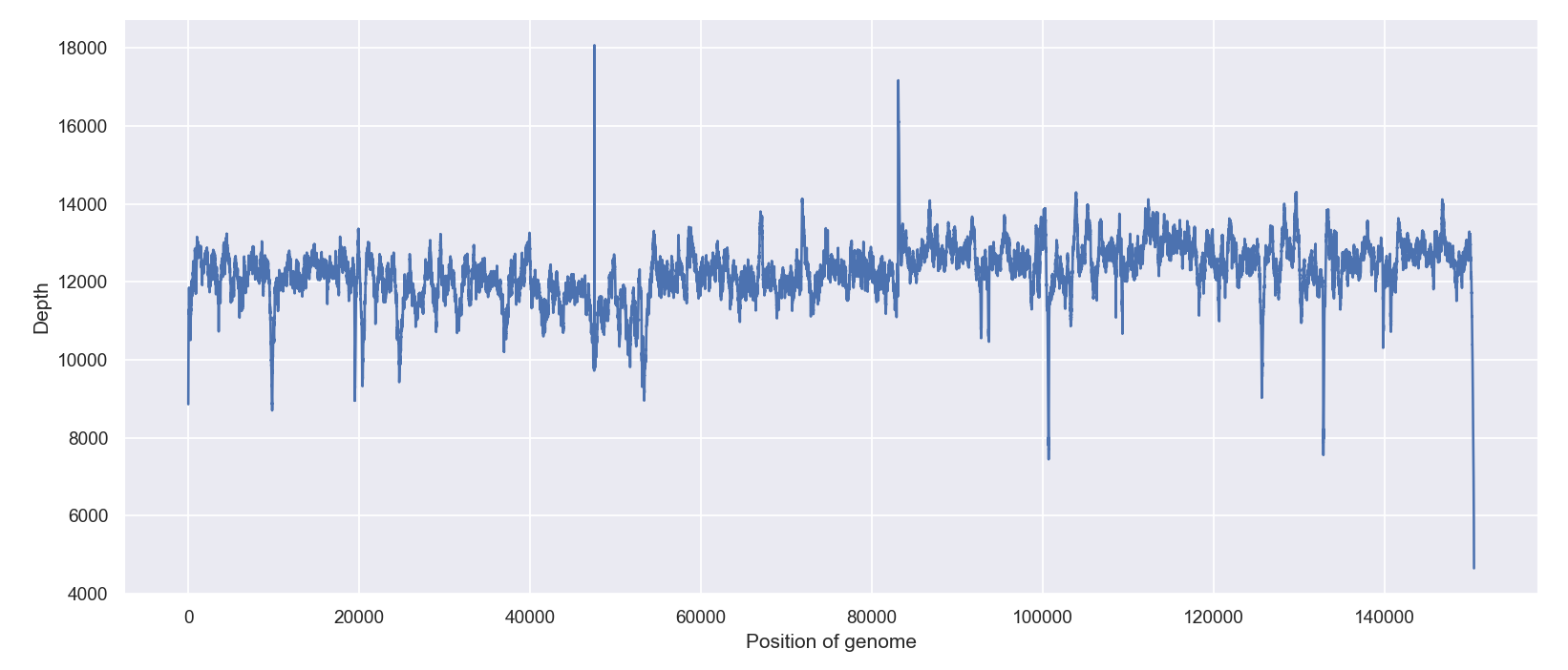


Figure S1. Chloroplast genome sequencing depth distribution for *Calendula officinalis.* The graph illustrates the chloroplast genome sequencing depth distribution for species *Calendula officinalis*, with the horizontal axis representing genomic position and the vertical axis indicating sequencing depth.


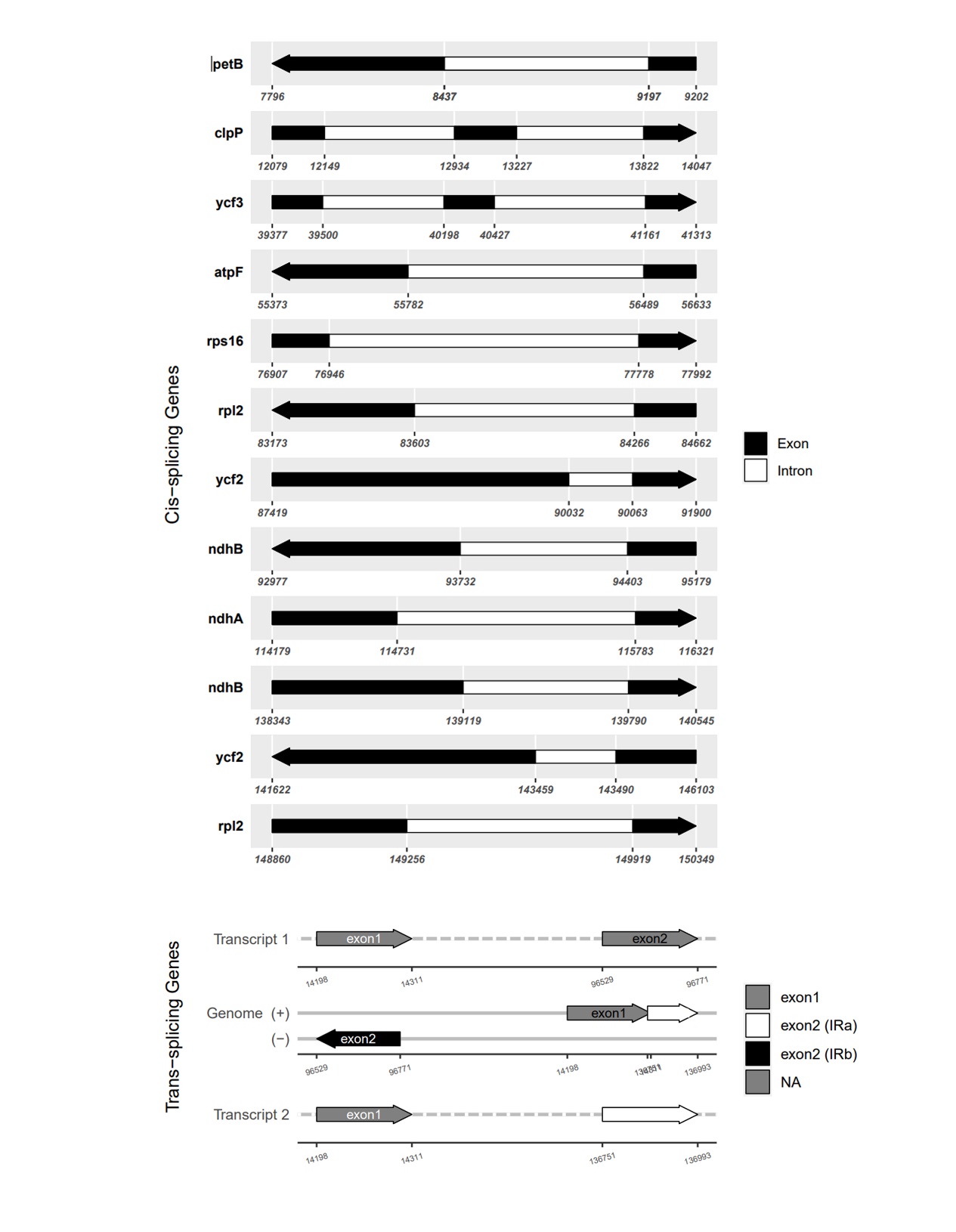


Figure S2. Schematic map of the cis-splicing genes and trans-splicing gene *rps12* in the chloroplast genome of *Calendula officinalis* using CPGView. The exons of the cis-splicing genes are shown in black; the introns are shown in white. The arrow indicates the sense direction of the gene. Please note that lengths of exons and introns are not drawn to scale.
